# Trends and determinants of complementary feeding practices in Tanzania, 2004–2016

**DOI:** 10.1186/s41182-018-0121-x

**Published:** 2018-11-20

**Authors:** Felix Akpojene Ogbo, Pascal Ogeleka, Akorede O. Awosemo

**Affiliations:** 1 Translational Health Research Institute (THRI), School of Medicine, Western Sydney University, Campbelltown Campus, Locked Bag 1797, Penrith, NSW 2571 Australia; 2 Prescot Specialist Medical Centre, Welfare Quarters, Makurdi, Benue State Nigeria

**Keywords:** Complementary feeding, Determinants, Malnutrition, Tanzania, Infant and young child, Trends

## Abstract

**Background:**

Following the successful implementation of the Millennium Development Goals (MDGs) strategy in Tanzania, improvements in child health indicators were observed. However, it remains unclear whether complementary feeding practices have improved given the renewed global agenda on child nutrition. This study investigated trends and socioeconomic and health service factors of complementary feeding practices in Tanzania for the period spanning from 2004 to2016.

**Methods:**

The study was based on the Tanzania Demographic and Health Survey data for the years 2004–2005 (*n* = 2480), 2010 (*n* = 2275) and 2015–2016 (*n* = 2949) to estimate the trends in complementary feeding practices. Multivariate logistic regression models that adjusted for year of the survey, clustering and sampling weights were used to investigate the association between the modifiable study factors (socioeconomic and health service factors) and complementary feeding practices among children aged 6–23 months in Tanzania.

**Results:**

Over the study period, minimum dietary diversity (MDD) and minimum acceptable diet (MAD) have worsened from 46% (95% confidence interval [95% CI] 41.5–50.7%) in 2004–2005 to 30% (95% CI 25.7–32.9%) in 2015–2016 and 16.9% (95% CI 14.9–18.9%) in 2004–2005 to 6.0% (95% CI 4.9–7.1%) in 2015–2016, respectively. Minimum meal frequency (MMF) remained unchanged, 37% in 2004–2005 and 2015–2016. The introduction of solid, semi-solid and soft foods improved from 79% (95% CI 74.5–83.9%) in 2004–2005 to 87% (95% CI 83.7–90.9%) in 2015–2016. Multivariate analyses revealed that higher maternal education and household wealth, mother’s employment, health facility birthing and postnatal care (PNC) visit were associated with MDD, MAD and MMF. Traditional birth attendant-assisted births and PNC visits were associated with the introduction of complementary foods. In contrast, birthing in the health facility was associated with the delayed introduction of complementary foods.

**Conclusion:**

Between 2004 and 2016, the prevalence and determinants of complementary feeding practices varied in Tanzania. Improving complementary feeding practices is feasible in Tanzania given the renewed focus on child nutrition in the country. Child nutrition policy interventions should target all mothers, particularly mothers from low socioeconomic background and those with limited access to health services to maximise results.

**Electronic supplementary material:**

The online version of this article (10.1186/s41182-018-0121-x) contains supplementary material, which is available to authorized users.

## Background

Exclusive breastfeeding (EBF) for infants aged less than 6 months is essential for growth and development; however, the breast milk alone is not adequate to meet the nutritional needs of the infant after the age of 6 months, indicating the need to introduce complementary foods [1]. The World Health Organization (WHO) recommends complementary feeding, defined as the introduction of nutritionally appropriate and safe foods for infants aged 6 months and above to supplement breastfeeding [1, 2]. Appropriate complementary feeding practices reduce the risk of childhood undernutrition (i.e. stunting, wasting and being underweight) [3], diarrhoea [4–6] and under-five mortality [3]. Despite the demonstrated benefits of appropriate complementary feeding practices, inappropriate complementary feeding is still widespread in many developing countries [7–9], including Tanzania [10, 11], with subsequent negative impact on child health and survival [3].

In Tanzania, complementary feeding practices are suboptimal as they do not meet the four WHO-recommended complementary feeding indicators (introduction of solid, semi-solid and soft foods; minimum dietary diversity; minimum meal frequency; and minimum acceptable diet) [12]. This is despite the adoption of the Global Strategy on Infant and Young Child Feeding (IYCF) [12] and the implementation of the Millennium Development Goal (MDG) strategies to improve child survival [13]. Previous studies based on the 1999 and 2010 nationwide surveys in Tanzania had elucidated some factors associated with inappropriate complementary feeding practices [10, 11]. These factors included the child’s age (6–11 months), limited access to mass media, lower level of paternal and maternal education, poor socioeconomic status and the paucity of postnatal check-ups. However, no nationally representative studies have examined trends in complementary feeding practices nor has there been an investigation of socioeconomic and health service factors associated with complementary feeding practices during the MDG period (2000–2015) in Tanzania. The exposition of trends in complementary feeding in Tanzania would show which complementary feeding practices are improving or decreasing in their distribution. This information is required to identify issues relating to complementary feeding practices and also to inform the effectiveness of current IYCF interventions.

Notably, Tanzania was one of the few countries that met the key MDG indicators (such as reduction in under-five mortality) globally [13, 14]. Investigating the trends and factors associated with complementary feeding practices would be essential to policy decision-makers and nutrition experts to provide targeted interventions. Similarly, findings from this study will also be of high importance to public health experts in Tanzania given the current United Nations Decade of Action on Nutrition (2016–2025) [15] and Sustainable Development Goal-2 (SDG-2) agenda, which has a strong global political commitment to end malnutrition in all its forms by 2030 [16]. This study aimed to investigate the trends and socioeconomic and health service determinants of complementary feeding practices in Tanzania between 2004 and 2016.

## Method

### Data source

This study utilised data from the Tanzania Demographic and Health Survey (TDHS) for the years 2004–2005, 2010 and 2015–2016, which were collected by the Tanzania National Bureau of Statistics (NBS), Dar es Salaam, in collaboration with the US Inner City Fund (ICF) International, MD. The TDHS provides comprehensive information on complementary feeding practices, and other child and maternal socioeconomic and health characteristics, obtained from a nationally representative sample of households. During the surveys, a stratified two-stage cluster design was used to select the samples, and standardised face-to-face questionnaires were administered to eligible women of childbearing age (15–49 years). The number of households selected in the surveys included *N* = 10,312 in 2004–2005, *N* = 9623 in 2010 and *N* = 13,360 in 2015–2016, with response rates that ranged from 96 to 98.8%. Further details on the data source, including sampling technique and method of data collection, are described in the respective TDHS reports [12, 17, 18].

In accordance with the TDHS approach [12], the WHO definitions for assessing complementary feeding practices in populations [2] and previously published studies [7, 10, 19], the analyses were restricted to the youngest living child aged 0–23 months, living with respondents (women aged 15–49 years). A weighted total sample of 7705 eligible women who met the complementary feeding criteria was used for the study (that is, *n* = 2480 for 2004–2005, *n* = 2275 for 2010 and *n* = 2949 in 2015–2016).

### Outcome variables

The outcome variables were the complementary feeding indicators (introduction of solid, semi-solid or soft foods; minimum dietary diversity; minimum meal frequency; and minimum acceptable diet), measured using the WHO definition for assessing IYCF practices [2].

- Introduction of solid, semi-solid or soft foods: The proportion of infants 6–8 months of age who received solid, semi-solid or soft foods.
- Minimum dietary diversity (MDD): The proportion of children 6–23 months of age who received foods from four or more food groups. The seven food groups used for tabulation of this indicator were grains, roots and tubers; legumes and nuts; dairy products (milk, yoghurt, cheese); flesh foods (meat, fish, poultry and liver/organ meats); eggs; vitamin A-rich fruits and vegetables; and other fruits and vegetables.
- Minimum meal frequency (MMF): The proportion of breastfed and non-breastfed children 6–23 months of age, who received solid, semi-solid or soft foods (including milk feeds for non-breastfed children); the minimum number of times or more (i.e. two times for breastfed infants 6–8 months, three times for breastfed children 9–23 months and four times for non-breastfed children 6–23 months in the previous day). “Meals” include both meals and snacks (other than trivial amounts), and frequency is based on caregiver report.
- Minimum acceptable diet (MAD): The proportion of children 6–23 months of age who received both *minimum dietary diversity* and *minimum meal frequency*. All indicators were based on a 24-h recall of the infant’s dietary intake by the mother.

### Exposure variables

The selection of the study factors was based on evidence from previous studies conducted in sub-Saharan Africa and South Asia [7, 10, 20–22], where modifiable socioeconomic and health service factors were associated with inappropriate complementary feeding practices. Those studies also suggested that these modifiable factors are essential in formulating specific policy efforts to improve complementary feeding practices. The socioeconomic factors included mother’s and father’s highest educational level, household wealth index and employment status. Health service factors included the delivery assistance, place of delivery, the number of antenatal and postnatal clinic visits.

### Analytic strategy

The analysis followed a similar approach employed in previously published studies [23, 24]. First, initial analyses involved the description of the study factors, the estimation of a series of frequencies and cross-tabulations to estimate the prevalence of complementary feeding practices from 2004 to 2016 and by the study factors (socioeconomic and health service variables). Second, univariate logistic regression analyses were employed to examine the probable factors associated with complementary feeding practices. Third, only those variables with a *P* value < 0.1 were entered into the multivariate models that adjusted for confounders (maternal marital status, sex of the baby, birth order and interval, geographical region, place of residence and maternal age) to investigate the factors associated with complementary feeding practices in Tanzania. In the models of socioeconomic factors, an additional adjustment was made for health service factors as confounders of the association between socioeconomic factors and complementary feeding indicators. A similar strategy was used in models of health service factors, where additional adjustment for socioeconomic factors was performed. The regression analyses were conducted separately for both the year-specific data (i.e. 2004–2005, 2010, and 2015–2016) and the combined dataset to examine any differences in the determinants.

Additionally, the TDHS data from 2004 to 2016 were combined in the analyses for the following reasons: (i) to assess the trends and modifiable determinants of complementary feeding practices during the MDG era in order to provide specific and relevant complementary feeding information to help policy decision-makers during the implementation of the United Nations action on nutrition and SDG-2 agenda; (ii) to create a unique opportunity to compare complementary feeding outcomes over time and (iii) to increase the statistical power of the study. In the models, adjustment for year of the survey in the combined dataset, as well as cluster variations and sampling weights in both the year-specific data and combined dataset, was conducted [25, 26]. The weighted frequencies, the prevalence of complementary feeding practices by year, the odds ratios (OR) and their corresponding 95% confidence intervals for both the year-specific data and the combined dataset were reported. All analyses were conducted in Stata, version 15.0 (StataCorp, College Station, TX, USA).

### Ethics

The national health research body in Tanzania (the Medical Research Coordinating Committee, MRCC) granted Measure DHS/ICF International granted the ethical approval to conduct the surveys. The questionnaires used during the surveys were approved by the ICF International Institutional Review Board (IRB) to ensure they met the US Department of Health and Human Services regulations for the protection of human participants as well as the host country’s IRB, to ensure compliance with national laws. The data used are available to apply for online, and approval was obtained from Measure DHS/ICF International for this analysis.

## Results

### Characteristics of the study participants

Between 2004 and 2016, the majority of mothers were employed while many mothers had no schooling. Many mothers had four or more ANC visits from 2004 to 2016. In contrast, many mothers made no PNC visits over the study period **(**Table 1**)**.

**Table 1:** Characteristics of the study population

	2004–2005 (*N* = 2480)		2010 (*N* = 2275)		2015–2016 (*N* = 2949)	
	*n*	%	*n*	%	*n*	%
Socioeconomic factors						
Mother’s employment						
Not working	349	14.1	301	13.2	656	22.3
Working	2130	85.9	1974	86.8	2293	77.7
Mother’s education						
No schooling	652	26.3	573	25.2	575	19.5
Primary education	1711	68.9	1522	66.9	1865	63.3
Secondary and above education	116	4.8	180	7.9	507	17.2
Father’s education						
No schooling	432	18.6	381	18.0	330	13.7
Primary education	1716	73.6	1526	71.8	1653	68.5
Secondary and above education	183	7.8	217	10.2	430	17.8
Household wealth						
Poor	1121	45.2	1033	45.4	1334	45.2
Middle	996	40.2	890	39.2	1107	37.5
Rich	362	14.6	351	15.4	509	17.3
Health service factors						
Place of delivery						
Home	1293	52.1	1106	48.6	1001	34.0
Health facility	1187	47.9	1169	51.4	1948	66.0
Postnatal visits						
None	2282	92.1	1512	66.4	1860	63.1
0–2 days	150	6.0	549	24.2	122	4.1
3–42 days	46	1.9	214	9.4	966	32.8
Antenatal visits						
None	78	3.1	60	2.6	74	2.5
1–3	927	37.3	1257	55.2	1392	47.2
4+	1473	59.6	958	42.1	1482	50.3
Delivery assistance						
Health professional	1151	48.6	1147	51.4	385	40.8
Traditional birth attendance	217	9.1	282	12.6	5	0.6
Other untrained personnel	999	42.2	803	36.0	551	58.6

*n* and % are the weighted count and proportion for each variable, respectively

### Trends in complementary feeding practices in Tanzania, 2004–2016

**Fig. 1:**
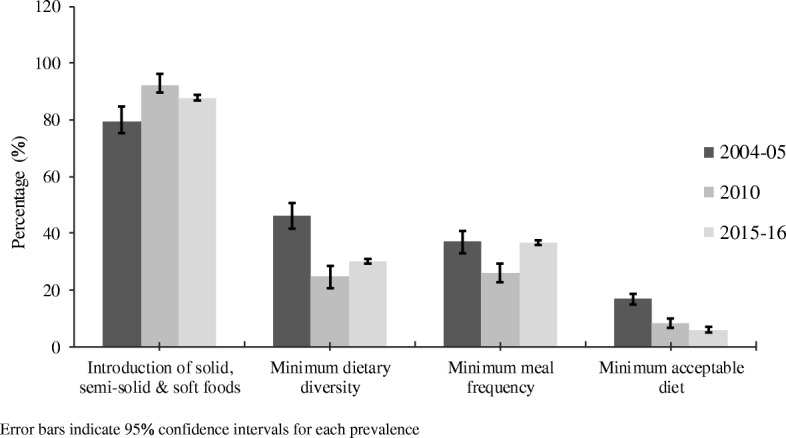
Prevalence of complementary feeding practices in Tanzania, 2004–2016

The proportion of mothers who introduced solid, semi-solid and soft foods to infants aged 6–8 months increased from 79% (95% confidence interval [95% CI] 74.5–83.9%) in 2004–2005 to 92% (95% CI 88.4–94.9%) in 2010 but worsened slightly to 87% (95% CI 83.7–90.9%) in 2015–2016 (Fig. 1). Between 2004 and 2016, a significant declining trend was observed among Tanzanian children who met the MDD and MAD from 46% (95% CI 41.5–50.7%) in 2004–2005 to 30% (95% CI 25.7–32.9%) in 2015–2016 and 16.9% (95% CI 14.9–18.9%) in 2004–2005 to 6% (95% CI 4.9–7.1%) in 2015–2016, respectively. However, MMF remained unchanged over the study period (37.0%, 95% CI 33.1–41.1%) in 2004–2005 and 2015–2016, despite a significant decrease in 2010 (26.2%, 95% CI 23.0–29.5%) (Fig. 1).

### Determinants of complementary feeding practices

#### Introduction of solid, semi-solid or soft foods

Mothers who delivered their babies in the health facility were less likely to introduce solid, semi-solid or soft foods to infants aged 6–8 months compared to those who delivered at home between 2004 and 2016 (Table 2). Receiving delivery assistance from a traditional birth attendant (TBA) was associated with delayed introduction of solid, semi-solid or soft foods compared to receiving assistance from health professionals. Mothers who had an early postnatal clinic (PNC, within 0–2 days) visit were more likely to introduce solid, semi-solid and soft foods to their infants aged 6–8 months compared to those who made no PNC visits over the study period. The year-specific data analyses showed no significant association between the study factors and the introduction of solid, semi-solid and soft foods (Additional file 1: Table S1).

**Table 2:** Determinants of introduction of solid, semi-solid and soft foods and minimum dietary diversity in Tanzania, 2004–2016

	Introduction of solid, semi-solid and soft foods (6–8 months, *n* = 1349)					Minimum dietary diversity (*n* = 7705)				
	%*	COR (95% CI)	*P* value	AOR* (95% CI)	*P* value	%*	COR (95% CI)	*P* value	AOR* (95% CI)	*P* value
Socioeconomic										
Mother’s employment										
Not working	84.5	1.00		1.00		25.4	1.00		1.00	
Working	86.7	1.28 (0.88–1.87)	0.194	1.64 (0.90–2.97)	0.100	26.8	1.11 (0.96–1.28)	0.131	1.21 (1.10–1.49)	0.029
Mother’s education										
No schooling	85.0	1.00		1.00		19.7	1.00		1.00	
Primary education	86.7	1.09 (0.74–1.62)	0.645	1.24 (0.73–2.11)	0.414	26.5	1.57 (1.36–1.82)	< 0.001	1.28 (1.06–1.53)	0.007
Secondary and higher	86.6	1.54 (0.86–2.75)	0.138	1.51 (0.56–4.06)	0.409	41.7	3.27 (2.71–3.94)	< 0.001	1.57 (1.17–2.08)	0.002
Father’s education										
No schooling	82.2	1.00		1.00		20.01	1.00		1.00	
Primary education	86.9	1.42 (0.93–2.16)	0.096	1.27 (0.71–2.29)	0.408	26.1	1.57 (1.32–1.87)	< 0.001	1.06 (0.87–1.31)	0.519
Secondary and higher	85.5	1.74 (0.99–3.07)	0.053	1.50 (0.60–3.77)	0.381	40.2	3.04 (2.47–3.75)	< 0.001	1.36 (1.03–1.81)	0.029
Household wealth										
Poor	86.2	1.00		1.00		18.7	1.00		1.00	
Middle	86.5	1.07 (0.74–1.53)	0.712	0.93 (0.56–1.56)	0.803	27.3	1.72 (1.51–1.95)	< 0.001	1.35 (1.16–1.59)	< 0.001
Rich	86.5	1.12 (0.74–1.53)	0.614	0.46 (0.17–1.20)	0.114	47.1	4.09 (3.48–4.79)	< 0.001	3.20 (2.48–4.14)	< 0.001
Health service										
Place of delivery										
Home	84.5	1.00		1.00		20.8	1.00		1.00	
Health facility	87.5	1.29 (0.92–1.80)	0.128	0.36 (0.47–0.92)	0.033	31.1	1.95 (1.73–2.18)	< 0.001	1.74 (1.29–2.34)	< 0.001
Postnatal visits										
None	84.5	1.00		1.00		26.6	1.00		1.00	
0–2 days	92.7	1.42 (0.81–2.48)	0.211	2.86 (1.21–6.72)	0.016	29.3	1.78 (1.38–2.29)	< 0.001	1.24 (0.92–1.67)	0.149
3–42 days	88.1	0.94 (0.51–1.76)	0.880	0.74 (0.26–2.13)	0.584	24.6	1.82 (1.41–2.35)	< 0.001	1.33 (1.00–1.79)	0.052
Antenatal visits										
None	89.5	1.00		1.00		17.8	1.00		1.00	
1–3	86.6	1.13 (0.42–3.04)	0.797	1.74 (0.56–5.37)	0.334	23.2	1.51 (0.99–2.30)	0.051	1.28 (0.80–2.05)	0.302
4+	85.6	1.25 (0.46–3.36)	0.651	1.34 (0.43–4.12)	0.607	30.2	1.99 (1.31–3.03)	0.001	1.40 (0.88–2.25)	0.152
Delivery assistance										
Health professional	87.6	1.00		1.00		35.9	1.00		1.00	
Traditional birth attendance	81.7	0.46 (0.25–0.87)	0.017	0.40 (0.18–0.87)	0.022	22.7	0.50 (0.41–0.62)	< 0.001	1.21 (0.85–1.73)	0.282
Other untrained personnel	85.0	0.81 (0.52–1.25)	0.347	0.90 (0.53–1.50)	0.703	22.8	0.56 (0.48–0.64)	< 0.001	1.27 (0.95–1.71)	0.098

%* is the proportion of weighted cases by the study factors. *COR* crude odds ratio; *AOR* adjusted odds ratio: adjusted for maternal marital status, sex of the baby sex and gender, birth order and interval, geographical region, place of residence and maternal age. In the models of socioeconomic factors, an additional adjustment was made for health service factors as confounders of the association between socioeconomic factors and complementary feeding indicators. A similar strategy was used in models of health service factors, where additional adjustment for socioeconomic factors was performed

#### Minimum dietary diversity

Between 2004 and 2016, children whose mothers had primary and higher educational level and those from wealthier households (middle and higher household wealth category) were more likely to meet MDD compared to those whose mothers had no schooling and from poorer households, respectively (Table 2). Similarly, children whose mothers were employed, those who birthed in a health facility and had PNC visit between 3 and 42 days were more likely to meet MDD compared to mothers with no employment, those whose mothers birthed at home and had no PNC visit, respectively (Table 2). Father’s higher education (secondary and higher) was associated with meeting the MDD compared to those whose fathers had no education. In the year-specific analyses, children whose mothers had higher educational attainment and those from wealthier households were more likely to meet MDD compared to those whose mothers had no education and those from poorer households, respectively (Additional file 2: Table S2). Similarly, children whose mothers birthed in a health facility were more likely to meet MDD compared to those whose mothers birthed at home, but the association was not evident in 2010.

#### Minimum meal frequency

The study showed that children whose mothers had secondary and higher educational level, those whose mothers were employed and those from richer households were more likely to meet MMF compared to those whose mothers had no schooling, were not in employment and were from poorer households from 2004 to 2016, respectively. Children whose mothers who made PNC visits were more likely to meet MMF compared to those whose mothers made no PNC visits (Table 3). The year-specific analyses showed that children whose mothers had early PNC (0–2 days) visits had higher odds for meeting MMF compared to those whose mothers had no PNC visits (Additional file 3: Table S3).

**Table 3:** Determinants of minimum meal frequency and minimum acceptable diet in Tanzania, 2004–2016

	Minimum meal frequency (*n* = 7705)					Minimum acceptable diet (*n* = 7705)				
	%*	COR (95% CI)	*P* value	AOR* (95% CI)	*P* value	%*	COR (95% CI)	*P* value	AOR (95% CI)	*P* value
Socioeconomic										
Mother’s employment										
Not working	37.1	1.00		1.00		8.3	1.00		1.00	
Working	38.8	1.11 (0.98–1.25)	0.098	1.31 (1.10–1.56)	0.002	10.5	1.33 (1.09–1.64)	0.005	1.48 (1.14–1.93)	< 0.001
Mother’s education										
No schooling	36.8	1.00		1.001.00		7.9	1.00		1.00	
Primary education	38.7	1.13 (1.00–1.27)	0.036	1.05 (0.90–1.23)	0.518	10.5	1.45 (1.18–1.78)	< 0.001	1.19 (0.92–1.53)	0.168
Secondary and higher	41.6	1.49 (1.27–1.76)	< 0.001	1.44 (1.11–1.87)	0.006	16.2	3.26 (2.53–4.20)	< 0.001	1.74 (1.19–2.55)	0.004
Father’s education										
No schooling	35.1	1.00		1.00		7.4	1.00		1.00	
Primary education	36.5	1.06 (0.92–1.22)	0.361	1.00 (0.84–1.20)	0.932	9.4	1.32 (1.04–1.67)	0.022	0.91 (0.69–1.21)	0.541
Secondary and higher	40.8	1.38 (1.16–1.65)	< 0.001	1.01 (0.78–1.31)	0.899	15.8	2.63 (1.99–3.48)	< 0.001	1.00 (0.69–1.46)	0.966
Household wealth										
Poor	35.0	1.00		1.00		6.9	1.00		1.00	
Middle	37.2	1.08 (0.97–1.20)	0.126	1.11 (0.96–1.29)	0.148	9.8	1.54 (1.29–1.85)	< 0.001	1.26 (1.00–1.58)	0.045
Rich	41.2	1.39 (1.21–1.61)	< 0.001	1.33 (1.02–1.73)	0.032	17.5	3.27 (2.65–4.03)	< 0.001	2.52 (1.77–3.59)	< 0.001
Health service										
Place of delivery										
Home	35.0	1.00		1.00		7.2	1.00		1.00	
Health facility	38.3	1.27 (1.15–1.40)	< 0.001	1.00 (0.75–1.33)	0.966	11.7	2.02 (1.72–2.38)	< 0.001	1.64 (1.04–2.58)	0.030
Postnatal visits										
None	40.0	1.00		1.00		9.6	1.00		1.00	
0–2 days	32.4	1.30 (1.14–1.49)	< 0.001	1.33 (1.13–1.57)	0.001	11.7	1.45 (1.17–1.79)	< 0.001	1.43 (1.11–1.84)	0.005
3–42 days	35.0	1.11 (0.92–1.32)	0.247	1.38 (1.02–1.87)	0.033	6.7	1.23 (0.90–1.70)	0.189	1.47 (0.09–2.41)	0.122
Antenatal visits										
None	31.3	1.00		1.00		6.3	1.00		1.00	
1–3	35.0	1.12 (0.80–1.55)	0.499	1.37 (0.91–2.07)	0.129	8.1	1.68 (0.86–3.27)	0.125	1.41 (0.66–3.00)	0.371
4+	38.8	1.34 (0.96–1.86)	0.077	1.47 (0.97–2.22)	0.066	11.3	2.34 (1.20–4.51)	0.012	1.70 (0.86–3.61)	0.162
Delivery assistance										
Health professional	36.4	1.00		1.00		14.5	1.00		1.00	
Traditional birth attendance	40.4	0.67 (0.56–0.81)	< 0.001	0.84(0.63–1.17)	0.306	8.4	0.51 (0.38–0.69)	< 0.001	1.12 (0.68–1.85)	0.646
Other untrained personnel	36.4	0.79 (0.70–0.89)	< 0.001	0.96 (0.72–1.27)	0.779	8.0	0.59 (0.44–0.65)	< 0.001	1.15 (0.75–1.76)	0.499

%* is the proportion of weighted cases by the study factors. *COR* crude odds ratio; *AOR* adjusted odds ratio: adjusted for maternal marital status, sex of the baby sex and gender, birth order and interval, geographical region, place of residence and maternal age. In the models of socioeconomic factors, an additional adjustment was made for health service factors as confounders of the association between socioeconomic factors and complementary feeding indicators. A similar strategy was used in models of health service factors, where additional adjustment for socioeconomic factors was performed

#### Minimum acceptable diet

In the pooled dataset, children whose mothers had secondary and higher education, those who were employed and those from middle and richer households were more likely to meet MAD compared to those whose mothers had no schooling, were not in employment and were from poorer households, respectively (Table 3). Children whose mothers who had early PNC (0–2 days) visits and those who birthed in the health facility were more likely to meet the MAD compared to those whose mothers reported no PNC visits and birthed at home, respectively (Table 3). The year-specific data analyses showed no significant association between the study factors and MAD (Additional file 4: Table S4).

## Discussion

The prevalence of introduction of solid, semi-solid and soft foods improved between 2004 and 2010 but decreased slightly in 2015–2016. Notably, a worsening trend was evident for MDD and MAD among Tanzanian children from 2004 to 2016. In contrast, the prevalence of MMF remained unchanged over the study period, despite a significant drop in 2010. The determinants of each of the complementary feeding indicators varied widely in the year-specific data and the pooled dataset.

Globally, higher maternal education is one of the most important factors needed to improve maternal and child health [27], including child nutrition [25, 28, 29] as articulated in the SDG-4 agenda, which aims to ensure that all girls and boys complete quality primary and secondary education by 2030 [16]. Higher maternal education increases a mother’s employment opportunity and household decision-making process [30] and is associated with an increase in health service use and reduced risk of maternal death [31]. The present study found that higher maternal education was associated with MDD, MMF and MAD. Similarly, higher household wealth and maternal employment were associated with MDD, MMF and MAD, possibly indicating the strong association between higher maternal education, improved household wealth and employment status [32, 33]. Our findings suggest that Tanzania may not only improve complementary feeding practices if the SDG-4 agenda is achieved, but also has the potential to increase the broader child health measures. Drawing lessons from the successful implementation of the MDG would also be crucial in the rollout of the current national nutrition strategic plan and SDG agenda in Tanzania.

Birthing in the health facility has significant benefit for both the mother and infant, including increased likelihood of the mother to engage in optimal infant feeding behaviours [26]. However, the present study found that birthing in a health facility in Tanzania was associated with the delayed introduction of solid, semi-solid or soft foods to infants aged 6–8 months. This finding is inconsistent with similar national studies conducted in Nigeria [7] and five South Asian countries—Bangladesh, India, Nepal, Pakistan and Sri Lanka [22, 34], which found no association between place of delivery and introduction of solid, semi-solid or soft foods. In comparison to sub-Saharan African countries with high diarrhoea mortality, Tanzania had the highest EBF prevalence (63%) [26], where mothers were likely to continue EBF instead of introducing complementary foods at aged 6–8 months as recommended. This practice of EBF beyond 6 months may be a likely reason for why mothers delay the introduction of complementary foods to their infants in Tanzania [6]. Additional plausible reasons for why birthing in a health facility was associated with the delayed introduction of complementary foods may include a lack of knowledge of appropriate complementary feeding practices among healthcare workers or issues around staff training or poor information transfer between healthcare workers and mothers [35]. Our finding implies that nutrition experts in Tanzania need to design specific interventions (such as appropriate complementary feeding practice messages) for mothers who birth in a health facility, and policy decision-makers need to adopt evidence-based approaches to improve child nutrition training for healthcare workers.

The study revealed that an early PNC visit (0–2 days) was associated with the introduction of complementary foods, despite the marginal drop in the prevalence from 2010 to 2016. For MDD and MAD, the evidence was somewhat mixed, where 3–42 days and 0–2 days PNC visits were associated with MDD and MAD, respectively. In addition, any PNC visit was associated with an increased likelihood of children aged 6–23 months to meet the MMF. These findings are similar to previously published studies conducted in Nigeria [7], Bangladesh, India, Nepal, Pakistan and Sri Lanka [22, 34], where no PNC visit was associated with inappropriate complementary feeding practices. In Tanzania, postnatal care services for both the mother and her baby involve the assessment of the baby (e.g. for danger signs), provision of IYCF information to the mother, cord examination and other services, including temperature measurement for the newborn and promotion of immunisation [12, 17]. Between 2004 and 2015, there was an improvement in the number of women who made PNC visits in Tanzania [12, 17]. Nevertheless, this increase in post-birth visits to a healthcare professional did not translate to improvement in the prevalence of MDD and MAD in the present study. Policy interventions that target women during the early post-birth period may be very helpful in improving the proportion of children who receive safe and nutritionally balanced complementary foods in Tanzania.

Our study suggests that Tanzania’s gains in maternal and child health indicators during the MDG era were not reflected in the MDD and MAD given the declining trend in these outcome measures. In Tanzania (like many sub-Saharan African countries), developmental assistance for health (DAH) plays a significant role in financing relevant public health programmes [28, 36–38]. The decline in MDD and MAD over the study period may be attributed to a shift in DAH from maternal and child health interventions (including child nutrition efforts) to HIV/AIDS programmes in sub-Saharan African countries [28, 37, 38], which was reasonably appropriate given the high HIV/AIDS burden in those countries. The decline may also be due to the adverse weather conditions that contribute to low farm outputs, farmers’ poor post-harvest food management systems and subsequently food insecurity [39, 40]. The present study also found that TBA-assisted births were associated with early introduction of complementary foods, while health facility birthing was associated with an increased likelihood of meeting MDD and MAD. During the MDG period, TBA-assisted births improved compared to relative/friend-assisted births in Tanzania [12, 17] due to an array of regional and national strategic initiatives implemented by the Government of Tanzania, including the National Road Map Strategic Plan to Accelerate Reduction of Maternal, Newborn and Child Deaths in Tanzania [41]. However, whether these interventions may have translated into optimal complementary feeding practices in the country remains unclear. Additional studies that focus on the impact of those programmes and place of delivery on complementary feeding practices may be warranted to elucidate the extent to which the place of birth impacts complementary feeding practices in Tanzania.

Currently, Tanzania is well-positioned given the strong political commitment to achieve the United Nations nutrition targets and SDGs, as well as the dedication to improve complementary feeding practices among children aged 0–23 months, with likely impact on child undernutrition in the short and long term. This is possible because of the contextualisation of global health actions on child nutrition in the country. For example, in 2016, the Tanzanian Government introduced the National Multisectoral Nutrition Action Plan (NMNAP, 2016–2021), with the improvement of complementary feeding practices being part of the aim for the strategy [42]. Nevertheless, nutrition efforts in Tanzania must be designed to translate the strong political will into appropriately resourced and locally relevant interventions that also consider the availability and accessibility of local food types and health services to mothers [6, 42]. Also, studies that examine the nutritional contents of local food types, particularly in poorly resourced communities, may be needed to guide context-specific health promotion messages in Tanzania.

This study has several limitations. First, the outcome variables were measured based on self-reported information. This is a possible source of measurement bias (including social desirability) in which mothers may have erroneously recall when, how and with what the child (0–23 months) was fed during the survey, or they may respond to questions in a manner that satisfies the interviewer. Second, misclassification in the study factors may also have occurred, where, for example, the number of ANC visits may have been over- or underestimated. This could result in over- or underestimation of the association between the health service variable and complementary feeding indicators. Third, as with the analysis of cross-sectional data, the interpretation of the causality between the study factors and complementary feeding practices is impossible. Finally, our results may also be limited given that we were unable to adjust for all contextual factors (such as family dynamics, a cultural belief system for child feeding and health system characteristics) that might affect the complementary feeding practices. Despite these limitations, the study has strengths. We believe that selection bias may be less likely to be present in this study given the high response rates in the surveys (96–98.8%). The TDHS data were collected by trained personnel who used standardised questionnaires that allow comparability across time. Our study provides locally relevant evidence on trends and determinants of complementary feeding practices during the MDG period to guide renewed efforts to improve child nutrition in Tanzania.

## Conclusion

The current study indicated that MDD and MAD have worsened between 2004 and 2016, but MMF remained unchanged. The introduction of solid, semi-solid and soft foods improved marginally. Common determinants of complementary feeding practices included higher maternal education and household wealth, mother’s employment, health facility birthing, PNC visits and TBA-assisted births for the introduction of complementary foods. Our findings suggest that Tanzania could improve complementary feeding practices given the current nutrition policy interventions. However, the translation of those policy decisions into measurable nutrition achievements is essential to improve child health outcomes.

## Additional files

Additional file 1: **Table S1.** Introduction of solid, semi-solid and softs foods by socioeconomic and health service characteristics, Tanzania 2004–2016. (DOCX 21 kb)

Additional file 2: **Table S2.** Minimum dietary diversity by socioeconomic and health service characteristics, Tanzania 2004–2016. (DOCX 21 kb)

Additional file 3: **Table S3.** Minimum meal frequency by socioeconomic and health service characteristics, Tanzania 2004–2016. (DOCX 21 kb)

Additional file 4: **Table S4.** Minimum acceptable diet by socioeconomic and health service characteristics, Tanzania 2004–2016. (DOCX 21 kb)

## Abbreviations

EBF: Exclusive breastfeeding
ICF: Inner City Fund
IYCF: Infant and Young Child Feeding
MAD: Minimum acceptable diet
MDD: Minimum dietary diversity
MDG: Millennium Development Goal
MMF: Minimum meal frequency
NBS: National Bureau of Statistics
SDG: Sustainable Development Goal
TBA: Traditional birth attendant
TDHS: Tanzania Demographic and Health Survey
WHO: World Health Organization
