# Supplementary material for: Trends and determinants of complementary feeding practices in Tanzania, 2004–2016
Source: Trop Med Health. 2018 Nov 20;46:40. doi: 10.1186/s41182-018-0121-x (PMC6247732; doi:10.1186/s41182-018-0121-x)
Supplement: Supplementary file 1 — Table S1. Introduction of solid, semi-solid and softs foods by socioeconomic and health service characteristics, Tanzania 2004–2016. (DOCX 21 kb) [file 41182_2018_121_MOESM1_ESM.docx]

**Table S1: Introduction of solid, semi-solid and softs foods by socio-economic and health service characteristics, Tanzania 2004-2016**

|  | 2004-05 | Adjusted | | |  | 2010 | Adjusted | | |  | 2015-16 | Adjusted | | |  | P |
| --- | --- | --- | --- | --- | --- | --- | --- | --- | --- | --- | --- | --- | --- | --- | --- | --- |
|  | (%)* | OR (LCI-UCI) | | | P | (%)* | OR 95%(LCI-UCI) | | | P | (%)* | OR (LCI-UCI) | | | P | trend |
| ***Socioeconomic*** | |  |  |  |  |  |  |  |  |  |  |  |  |  |  |  |
| *Mother's employment* | | |  |  |  |  |  |  |  |  |  |  |  |  |  |  |
| Not working | 87.8 | 1.00 |  |  |  | 93.2 |  |  |  |  | 87.1 | 1.00 |  |  |  | 0.906 |
| Working | 89.5 | 1.56 (0.60-4.00) | | | 0.355 | 92.2 | 1.58 (0.48-5.14) | | | 0.446 | 88.8 | 1.22 (0.59-2.48) | | | 0.583 | 0.248 |
| *Mother's education* | | |  |  |  |  |  |  |  |  |  |  |  |  |  |  |
| No schooling | 85.6 | 1.00 |  |  |  | 89.1 | 1.00 |  |  |  | 86.1 | 1.00 |  |  |  | 0.320 |
| Primary education | 89.9 | 1.76 (0.88-3.5) | | | 0.109 | 94.2 | 1.04 (0.39-2.72) | | | 0.936 | 87.5 | 0.76 (0.31-1.83) | | | 0.543 | 0.163 |
| Secondary & above education | 99.0 | 2.38 (0.92-6.11) | | | 0.071 | 87.6 | 1.61 (0.31-8.39) | | | 0.569 | 90.6 | 0.35 (0.040-3.62) | | | 0.543 | 0.006 |
| *Father's education* | | |  |  |  |  |  |  |  |  |  |  |  |  |  |  |
| No schooling | 86.6 | 1.00 |  |  |  | 88.5 | 1.00 |  |  |  | 75.1 | 1.00 |  |  |  | 0.748 |
| Primary education | 88.1 | 1.55 (0.70-3.4) | | | 0.276 | 92.8 | 0.66 (0.45-3.52) | | | 0.661 | 90.1 | 1.51 (0.63-3.59) | | | 0.350 | 0.518 |
| Secondary & above education | 97.8 | 2.4 (0.55-9.99) | | | 0.242 | 91.6 | 2.80 (0.49-18.5) | | | 0.228 | 87.8 | 1.45 (0.51-4.09) | | | 0.479 | 0.452 |
| *Household Wealth* | | | |  |  |  |  |  |  |  |  |  |  |  |  |  |
| Poor | 89.6 | 1.00 |  |  |  | 90.3 | 1.00 |  |  |  | 85.5 | 1.00 |  |  |  | 0.877 |
| Middle | 87.0 | 0.88 (0.43-1.80) | | | 0.740 | 95.5 | 1.61 (0.74-4.64) | | | 0.366 | 90.9 | 1.13 (0.55-2.32) | | | 0.736 | 0.898 |
| Rich | 94.0 | 1.28 (0.31-4.54) | | | 0.701 | 90.9 | 3.91 (0.22-3.61) | | | 0.895 | 88.3 | 1.00 (0.40-2.47) | | | 0.993 | 0.295 |
| ***Health Service*** | |  |  |  |  |  |  |  |  |  |  |  |  |  |  |  |
| *Place of delivery* | |  |  |  |  |  |  |  |  |  |  |  |  |  |  |  |
| Home | 87.9 | 1.00 |  |  |  | 90.4 | 1.00 |  |  |  | 83.8 | 1.00 |  |  |  | 0.564 |
| Health facility | 90.6 | 1.32 (0.63-2.78) | | | 0.450 | 93.7 | 2.10 (0.82-5.39) | | | 0.120 | 89.8 | 1.84 (0.97-3.48) | | | 0.060 | 0.368 |
| *Postnatal visits* | | | |  |  |  |  |  |  |  |  |  |  |  |  |  |
| None | 78.7 | 1.00 |  |  |  | 89.5 | 1.00 |  |  |  | 88.4 | 1.00 |  |  |  | 0.215 |
| 0-2 days | 87.7 | 2.83 (0.26-30.0) | | | 0.746 | 95.5 | 2.10 (0.50-8.86) | | | 0.308 | 82.9 | 1.02 (0.80-13.7) | | | 0.984 | 1.000 |
| 3-42 days | 100 | 2.93 (0.47-31.4) | | | 0.845 | 100 | 2.20 (1.02-9.71) | | | 0.038 | 87.5 | 0.35 (0.08-1.42) | | | 0.146 | 0.722 |
| *Antenatal visits* | |  |  |  |  |  |  |  |  |  |  |  |  |  |  |  |
| None | 86.5 | 1.00 |  |  |  | 95.4 | 1.00 |  |  |  | 97.3 | 1.00 |  |  |  | 0.024 |
| 1-3 | 87.6 | 5.33 (0.96-29.37) | | | 0.054 | 94.4 | 2.58 (0.23-28.57) | | | 0.770 | 88.3 | 1.02 (0.65-1.62) | | | 0.922 | 0.988 |
| 4+ | 91.8 | 7.6 (1.39-41.61) | | | 0.019 | 89.2 | 1.03 (0.99-10.90) | | | 0.030 | 86.3 | 1.05 (0.71-1.53) | | | 0.817 | 0.722 |
| *Delivery assistance* | |  |  |  |  |  |  |  |  |  |  |  |  |  |  |  |
| Health professional | 79.9 | 1.00 |  |  |  | 93.5 | 1.00 |  |  |  | 91.3 | 1.00 |  |  |  | 0.488 |
| Traditional birth attendance | 68.0 | 0.02 (0.0008-0.48) | | | 0.016 | 94.6 | 0.31 (0.002-36.4) | | | 0.633 | 0.00 | 0.00 (0.00-0.00) | | | 0.000 | 0.129 |
| Other untrained personnel | 83.6 | 0.99 (0.008-1.23) | | | 0.073 | 89.1 | 0.39 (0.003-41.2) | | | 0.696 | 82.4 | 0.08 (0.001-6.58) | | | 0.266 | 0.495 |

(%)* = *proportion of weighted cases by the study factors; P trend = trend in each variable over the study period; Multivariable models adjusted for the potential confounding factors of maternal marital status, sex of the baby sex and gender, birth order and interval, geographical region, place of residence and maternal age.* *In models of socioeconomic factors, an additional adjustment was made for health service factors as confounders of the association between socioeconomic factors and complementary feeding indicators. A similar strategy was used in models of health service factors, where additional adjustment for socioeconomic factors was performed*
